# Studies on safety and efficacy of particles containing a mixture of hydroxyapatite–argentum–titanium oxide (HAT) and sheets coated with HAT particles to be used in masks to improve nasal allergy: II. Cellular, in vivo, and clinical studies

**DOI:** 10.1007/s00405-022-07289-8

**Published:** 2022-03-06

**Authors:** Narumi Okazaki, Dai Yamaki, Toshio Takei, Miyuki Shimizu, Naoyuki Kamatani, Takayuki Shindo

**Affiliations:** 1 DR.C Medical Medicine Co. Ltd., Shinjuku, Tokyo Japan; 2 Medical Lab Partners Corporation, Chiyoda, Tokyo Japan; 3 StaGen Co. Ltd., Taito, Tokyo Japan; 4 Department of Cardiovascular Research, Shinshu University School of Medicine, Matsumoto, Nagano Japan; 5 Department of Life Innovation, Institute for Biomedical Sciences, Interdisciplinary Cluster for Cutting Edge Research, Shinshu University, Matsumoto, Nagano Japan

**Keywords:** Hydroxyapatite, Titanium oxide, Nasal allergy, Mite allergy, Cedar pollinosis, Photocatalysis

## Abstract

**Purpose:**

We report the manufacture of particles containing a mixture of hydroxyapatite–argentum–titanium oxide (HAT), followed by attachment to nonwoven polyester fabrics to produce HAT-coated sheets (HATS) for use in masks. The purpose of the present study was to perform cellular, in vivo, and clinical studies to further examine the safety of HATS for use in masks to improve nasal allergy.

**Methods:**

Reverse mutation tests for HAT were performed using five bacterial strains. A cellular toxicity test was performed using a Chinese hamster cell line incubated with the HATS extracts. Skin reactions after intradermal administration were examined in rabbits. Skin sensitization tests in guinea pigs were performed using the HATS extracts. HAT was administered to the nasal cavity and conjunctival sac of the rabbits. An oral administration study was performed in rats. Finally, a human skin patch test was performed using the HATS.

**Results:**

Reverse mutation tests showed negative results. The cellular toxicity test showed that the HATS extract had moderate cytotoxicity. The intradermal skin reaction and **s**kin sensitization tests were all negative. The administration of HAT to the nasal cavity and intraocular administration showed negative results. No toxicity was observed after oral administration of HAT powder up to a dose of 2000 mg/kg. Finally, the skin patch test result was negative.

**Conclusion:**

Although HAT showed moderate cytotoxicity, in vivo results indicated that HAT is safe because it does not come in direct contact with cells in normal usage, and HATS is safe when used in masks.

## Introduction

After World War II, the Japanese cedar tree, an indigenous tree species, was planted in Japan according to government policy. As a result, approximately 4.5 million hectares are now covered by cedar plantations that occupy about 40% of the planted forests in Japan. Approximately 30% of the Japanese population is estimated to be affected by cedar pollinosis, and many Japanese people suffer from severe sneezing and nasal congestion between February and April. Cedar pollinosis has significant social and economic impacts. In addition to pollinosis, nasal allergy caused by dust is an important perennial allergy. Dust containing mite allergens is an important source of allergies.

Various attempts have been made to prevent or improve cedar pollinosis. Medicines are undoubtedly effective; however, there are also other ways of preventing or improving cedar pollinosis. For example, there are gargles and masks. Masks are expected to be effective in reducing the amount of pollen inhaled by one-third to one-sixth when pollen counts are high, thus reducing nasal symptoms. Furthermore, chemical modification of the mask material could have an even stronger improvement effect [1].

The photocatalytic action of titanium dioxide has a wide range of applications, including the purification and disinfection of water and air [2, 3]. Organic substances such as proteins are decomposed by the active oxygen species generated by the photocatalytic action of titanium dioxide. When titanium dioxide is irradiated with ultraviolet light, free radicals are generated [4]. However, when silver is added to titanium dioxide, it absorbs both ultraviolet light below 380 nm and visible light between 700 and 800 nm, which enhances the photocatalytic effect [5–8]. In contrast, hydroxyapatite (HA) can adsorb organic substances such as proteins and viruses [9]. Nonami et al. reported that HA coated with titanium dioxide effectively removes airborne materials [10]. The organic substances that cover the surface of HA reduce its adsorption capacity, but the reactive oxygen species generated by the titanium dioxide decompose the adsorbed organic substances, thereby improving the adsorption capacity of HA [10].

To prevent pollinosis and dust-induced nasal allergies, we devised a method of producing particles made of a mixture of HA, silver, and titanium oxide (HAT), which adsorb and decompose allergens [1]. We attached HAT particles to the non-woven fabrics used in masks. The HAT attached sheet (HATS) was placed between two sheets of non-woven fabrics and used as a mask to improve nasal allergies [1].

Allergens that cause nasal allergies are trapped by HAT particles as they pass through the mask and are expected to be efficiently broken down. In our previous study, we showed that Cryj1, a major allergen of cedar pollinosis, and Der f1, a major allergen of mite allergy, were efficiently reduced by HATS [1]. The reduction of these allergens by HATS was observed under light-shielded conditions, but the reduction was even greater under light irradiation, thereby suggesting the absorption of major nasal allergens by HATS particles and their decomposition by the photocatalyst. Therefore, masks with HATS interspersed between non-woven fabrics are expected to reduce allergens and alleviate nasal allergies before allergens are detected. However, because masks are worn over the nose and mouth for a long time, some of the HAT particles attached to the fabric may be exposed. Therefore, the safety of the HATS and HAT particles needs to be studied in detail.

In general, it has been reported that nanoparticles smaller than 100 nm can cause harm by oral ingestion [11, 12], inhalation [13], and skin contact [14]. We investigated the particle size distribution of HAT particles and their binding mode to non-woven fabrics using a laser scattering particle size analyzer and scanning electron microscopy [1]. As a result, the particles in our HAT powder showed a unimodal particle size distribution close to a lognormal distribution, with a median of 371.9 nm. Ninety-five percent of the particle sizes ranged from 199.8 to 1079.9 nm, and no particles smaller than 131 nm were detected [1]. Scanning electron microscopy showed that the HAT particles were attached to the fibers of the non-woven fabric as spherical and flattened materials [1].

Based on the above rudimentary safety data of HAT particles and HATS, various biological and medical tests were performed in this study. Here, we report the results of mutagenicity tests using bacteria, toxicity tests using cells, and in vivo experiments using animals. In addition, skin patch tests were conducted in a clinical trial, and safety data were obtained.

## Methods

### Reverse mutation tests using bacteria

Reverse mutation tests using HAT were conducted using five bacterial strains, *Salmonella typhimurium* TA98 and TA1537 with frame-shift mutations, TA100 and TA1535 [15], and *Escherichia coli* WP2 uvrA with single nucleotide substitutions [16] obtained from the Japan Bioassay Research Center (Hatano-shi, Japan). The procedures are essentially based on a previous report [17] and in accordance with the guidelines of the Japanese government (Guidelines for the Act on the Evaluation of Chemical Substances and Regulation of Their Manufacture, etc., 1997; Safety and Health Law Test Guidelines 1988, 1997; Guidelines for Biological Testing of Medical Devices 1995).

Briefly, after thawing, 20 mL of the cryopreserved bacterial suspension was added to 10 mL of liquid complete medium made of 25 g nutrient broth No. 2 (Oxid Unipath, Hampshire, UK) in 1 L distilled water and, after shaking the medium for 8 h at 37 °C, the bacteria in the medium were used for the culture. A 35% water suspension of HAT powder stored in the dark at room temperature (18–23 °C) was used as the test material. Then, a 0.1 mL solution containing varying concentrations of HAT powder in sterilized water was mixed with 0.5 mL of either 0.1 M NaPO_4_ (pH 7.4) or exogenous metabolic activation system S9 mix (Kikkoman Biochemifa Company, Tokyo, Japan), and 0.1 mL of the bacteria-containing solution. Thereafter, the combined suspension was fully mixed by shaking for 20 min at 37 °C, placed on Tesmedia AN medium (Oriental Yeast Industry; Tokyo, Japan), and cultured for 48 h at 37 °C. Then, the number of colonies was counted using an auto-colony counter or visual inspection.

### Cytotoxicity test using colony formation test

All procedures were performed in accordance with ISO 10993-5:2009(E) and “Japanese Drug and Food Safety Bureau Notification No. 0301-20, March 1, 2012: Basic Biological Safety Assessment Required for Application for Approval of Manufacture and Sale of Medical Devices”.

M05 medium was prepared by adding nonessential amino acids for minimum essential medium (ICN Biochemicals, Cranberry Township, PA), 0.11 g/L sodium pyruvate (ICN Biochemicals) and 5 vol% of fetal bovine serum (Sanko Junyaku in Tokyo) to Eagle MEM medium (Thermo Fisher Japan, Tokyo). Then, 35% HAT suspension in water was lyophilized and autoclaved, and 10 mL of M05 medium was added to 1 g of HAT. After incubating the HAT-containing medium for 24 h, only the medium was recovered and used as the 100% HAT extract. Polyurethane films containing 0.1% zinc diethyldithiocarbamate (standard material A), polyurethane film containing 0.25% zinc dibutyldithiocarbamate (standard material B), and high-density polyethylene film (negative control material) were obtained from the Food and Drug Safety Center (Hatano-shi, Japan) and cut into small pieces and autoclaved. Thereafter, the samples were treated as described for HAT, and the extracts were used as 100% extracts.

Cells from the V79 Chinese hamster cell line [18] obtained from RIKEN (Yokohama, Japan), cultured in M05, were used. Aliquots (5 mL) of the cell suspension (adjusted to 40 cells/mL) were seeded onto 60 mm plates and incubated in a CO_2_ incubator for 24 h at 37 °C. After removal of the culture medium, 5 mL of each extract was added to a plate. Three plates were used per concentration per extract (HAT, standard material A, standard material B, or negative control material). In addition, three plates to which only medium was added were used as negative control plates. After the addition of the extracts, the plates were incubated in a CO_2_ incubator for six days.

After incubation, the media were discarded from all plates, and the plates were washed with 5 mL of phosphate-buffered solution. Then, 5 mL of methanol was added to each plate and left for 10 min to fix the cells. After fixation, the methanol was discarded, and 5 mL of 5% Giemsa solution was added to the plates and incubated for 10 min. Plates with stained colonies were washed with sterile water. After colony staining, the number of colonies on each plate was counted under a microscope, and those with more than 50 cells were considered as colonies.

The colony formation rate (%) was calculated by dividing the average number of colonies for each extract concentration by the average number of colonies for the negative control plate and multiplying by 100. Based on the relationship between the concentration of the extract and the colony formation rates, the concentration of the extract that inhibits the number of colonies by 50% was determined using the probit model [19] and designated as IC50. If the colony formation rate exceeded 100%, the rate was considered to be 100%.

### Intradermal administration of the extract in rabbits

All animal studies described in this manuscript were performed in accordance with the Principles of Laboratory Animal Care (NIH publication No. 86-23, revised 1985), as well as specific Japanese guidelines, including “Basic Guidelines for the Implementation of Animal Experiments, etc. at Institutions under the Jurisdiction of the Ministry of Health, Labour and Welfare of Japan" established in 2006.

Two sheets of HATS (surface area of one sheet: 2 cm × 15 cm) were placed in an extraction vessel (glass bottle), and 20 mL of either saline or sesame oil (Wako Pure Chemical, Tokyo, Japan) was added to the vessel. Extraction was carried out at 120 °C for 1 h, and the extract without sheets was used after cooling to room temperature (18–23 °C). To prepare control extracts in saline and sesame oil, the same procedure as that used to prepare the HATS extract was performed except for the addition of HATS.

Four different extracts were used—HATS extract in saline, HATS extract in sesame oil, control in saline, and control in sesame oil. Each extract (0.2 mL) was injected at five points in the back of each rabbit.

In a separate experiment, extracts were prepared using non-woven fabric instead of HATS in saline and sesame oil, and the effect of the intradermal injection of the extracts of non-woven fabric was examined. The procedures were almost the same as those for the HATS extracts, except for the conditions for the extraction. Thus, in the procedure for the non-woven fabric, extraction was performed for 24 h at 70 °C.

The skin regions where the extracts were injected were observed at 24, 48, and 72 h after injection. Scoring for erythema and crusting was as follows: no erythema (0), mild erythema (1), obvious erythema (2), moderate to severe erythema (3), and severe erythema to slight crusting (4); edema scoring was as follows: no edema (0), very mild edema (1), mild edema (2), moderate edema (3), and severe edema (4).

### Skin sensitization test in guinea pigs

**Fig. 1:**
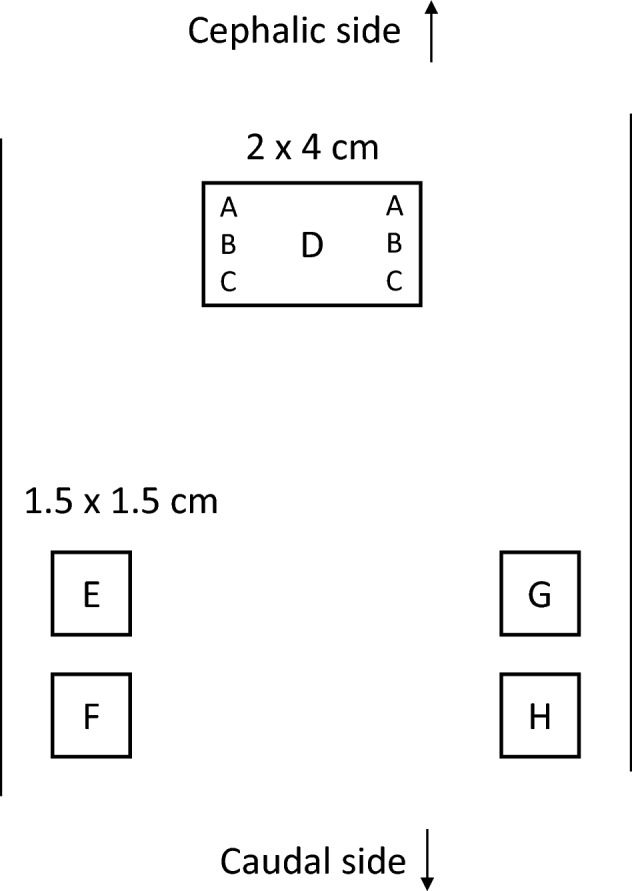
Sites of intradermal injection (**A**–**D**) and areas where lint cloth patches were attached (**E**–**H**) to the back of a guinea pig

A skin sensitization test was conducted on guinea pigs using saline and sesame oil extracts of HATS in accordance with ISO 10993-10. Extracts of HATS in saline and sesame oil as well as the control extracts were prepared according to the same procedure as for the previous experiment, that is, intradermal administration of the extract using rabbits. On day 0, 0.1 mL of the solutions indicated in Table 1 were intradermally injected at six sites (primary sensitization A, B, and C shown in Table 1) within a 2 × 4 cm square area (Fig. 1) at the back of animals for groups as described in Table 1.

**Table 1:** Skin sensitization test

Group	Primary sensitization			Secondary sensitization	Number of animals
	A	B	C	D	
Extract in saline	Water + FCA^a^	100% saline extract	100% saline extract + FCA	100% saline extract	10
Control in saline	Water + FCA	Saline control	Saline control + FCA	Saline control	5
Extract in sesame oil	Water + FCA	100% sesame oil extract	100% sesame oil extract + FCA	100% sesame oil extract	10
Control in sesame oil	Water + FCA	Sesame oil control	Sesame oil control + FCA	Sesame oil control	5
Positive control	Water + FCA	0.1% DNCB^b^	0.2% DNCB + FCA	0.1% DNCB	5

^a^Freund’s complete adjuvant

^b^1-Chloro-2,4-dinitrobenzene

On the day 7, a 2 × 4 cm filter paper permeated with the test solution shown in Table 1 (secondary sensitization) was attached to site D (Fig. 1), and the paper was occluded with polyethylene film tape for 48 h. On the 22nd day, a lint cloth patch permeated with the solution shown in Table 2 was attached to sites E, F, G, and H (Fig. 1) of the skin and occluded with polyethylene film tape for 24 h. Thereafter, skin reactions were observed according to the Magnusson and Kligman scale [20] at 24 and 48 h after patch removal.

**Table 2:** Induction test

Group	Inducing test			
	E	F	G	H
Extract in saline	100% saline extract	50% saline extract	25% saline extract	Saline control
Saline control				
Extract in sesame oil	100% sesame oil extract	50% sesame oil extract	25% sesame oil extract	Sesame oil control
Sesame oil control				
Positive control	0.1% DNCB	Acetone	–	–

### Administration of HAT to the nasal cavity of rabbits

The intranasal administration test in rabbits was performed in accordance with the OECD Guidelines for the Testing of Chemicals (No. 417, 2010). HAT powder was suspended in water (10 mg/mL weight/volume), and 50 mL of the suspension was administered intranasally to the left nasal cavity of each rabbit. The right nasal cavity was used as the control. Six animals were used and nasal mucosal samples were collected from three rabbits each, after 7 and 14 days of administration for pathological study (hematoxylin–eosin staining and toluidine blue staining). The plasma concentrations of the test substances (silver and titanium) were determined in three animals 7 days after administration. The concentrations of both silver and titanium were determined by inductively coupled plasma mass spectrometry (ICP-MS, ELAN DRC II, PerkinElmer SCIEX, No. 1). The mucosa near the entrance of the nasal cavity where the HAT suspension was administered was visually inspected daily, and the conditions of the mucosa were judged based on erythema and edema according to Draize scoring system for skin and mucosal lesions [21].

In a separate experiment, HAT was administered daily for 14 or 28 days. A 50-mL suspension of HAT powder (10 mg/mL, weight/volume) was administered daily to the left nasal cavity for 14 or 28 days to determine whether HAT has a cumulative effect on irritation of the nasal mucosa. The right nasal cavity was used as the control. Pathological examination of the samples collected from the nasal cavity was performed in three rabbits each for the 14-day and 28-day groups, as described in the single administration study. The plasma concentrations of the test substances (silver and titanium) were examined in three animals in the 28-day group.

### Intraocular administration study using rabbits

Intraocular administration tests in rabbits were performed according to the OECD Guideline for the Testing of Chemicals (No. 405, 2012). HAT suspension (0.1 mL) (34.5 weight %) was dropped once into the conjunctival sac of a total of three rabbits. Corneal, iris, and conjunctival changes in the treated eyes were observed grossly at 1, 24, 48, and 72 h after dosing, followed by detailed observation using a slit lamp, and judged according to the evaluation criteria described in the OECD guidelines [22]. The extent of corneal opacity and conjunctival effusion was judged according to Draize evaluation criteria [23]. Upon observation 24 h or later after the administration, after the observation using a slit lamp, approximately 0.1 mL of saline was added to the sodium fluorescein-containing portion of 0.7 mg Flores Eye Test Paper (Lot No. 6181 V, Ayumi Pharmaceutical Co., Ltd.) and the paper was attached to the cornea. After 2–3 s, the eye was rinsed with approximately 20 mL of purified water and checked for damage to the corneal epithelium using a slit lamp.

### Oral administration study using rats

The HAT suspension was prepared by mixing 35% HAT powder with 65% water. In the first experiment, a single dose of 2000 mg/kg of HAT was orally administered to five male and five female Sprague Dawley (SD) rats (obtained from Charles River, Yokohama, Japan) using a feeding tube, and they were observed for 14 days after administration. Animal death, general conditions, and weight were monitored daily. Necropsies were performed after the experiment.

In the next experiment, 36 male and 36 female rats were randomly assigned to 6 groups, each with 6 female and 6 male rats, for HAT powder doses of 0, 30, 100, 300, 1000, and 2000 mg/kg. HAT powder was mixed with water in a syringe and orally administered to each rat. For 14 days after the administration, animal death was checked at least once a day, and body weight was measured on Day 1 (before dosing), Day 4, Day 8, and Day 15; food intake was measured on Days 1, 4, 8, and 15 (before the end of the study), and both gross necropsy and histological examinations were performed on Day 15, followed by hematoxylin–eosin staining and microscopic examination.

### Human patch test using HATS

The human patch test was conducted by the Japan Hair Science Association (Tokyo, Japan), a contract testing organization. Written informed consent was obtained from each subject, and the study was approved by the Ethics Committee of the Japan Hair Science Association (Tokyo, Japan) and conducted in accordance with the ethical standards of the Declaration of Helsinki. Details that might disclose the identities of the subjects under study were omitted.

A small piece of HATS, cut into a 10 × 10 mm area, was applied to the flexor side of the subject’s upper arm using a tape for Finn Chamber (EPITEST Ltd., Fessenheim, France). The sample was removed 48 h after the application, and the skin was observed and judged by a dermatologist at 1 h and 24 h after removal.

The following International Contact Dermatitis Research Group (ICDRG) criteria were used for judgment [24]:

Negative: − (no reaction), ± (faint erythema only).

Positive: + (erythema, infiltrates, possibly papules), ⧺ (erythema, infiltrates, papules, vesicles), ⧻ (bullae).

## Results

### Reverse mutation test using bacteria

Five different bacterial strains, *Salmonella typhimurium* TA98, TA1537, TA100, TA1535, and *Escherichia coli* WP2 uvrA, were used to perform reverse mutation tests as described in the Methods section. Culturing was performed for each strain in duplicate plates, and the same experiment was performed twice. The number of revertant colonies induced by the test substance in the absence or presence of S9 mix never exceeded twice the control value for each test bacterium, and thus the mutagenicity of HAT was negative.

### Cytotoxicity test using colony formation

Cytotoxicity tests using colony formation were performed using V79 cells obtained from a Chinese hamster cell line. In a preliminary study using an extract of non-woven fabrics without HAT, no decrease in the colony formation rate was observed, indicating that the non-woven fabrics were not cytotoxic. Then, HAT extract, standard material A (polyurethane film containing 0.1% zinc diethyldithiocarbamate), standard material B (polyurethane film containing 0.25% zinc dibutyldithiocarbamate), and negative control material (high-density polyethylene film) were tested. V79 cells were cultured with various concentrations of HAT extract, and the colony formation rate decreased with increasing concentrations of the extract (Table 3). The concentration of the test substance that inhibited the number of colonies formed by V79 cells by 50% (IC50) was 53.5% (95% CI 47.3–63.0%). Data from standard materials A and B showed a dose-dependent inhibition of colony formation (Table 3). Based on these results, the HATS extract, but not the non-woven fabric extract, had moderate cytotoxicity.

**Table 3:** Cytotoxicity test

Test material	Concentration of extract (%)	Mean colony count	Colony forming rate (%)	IC50 (%) (95% CI)
HAT extract	100	0	0	53.5 (47.3–63.0)
	33.3	147.3	90.5	
	11.1	160.0	98.3	
	3.7	169.3	100	
Standard A^a^	5	0	0	2.3 (2.1–2.4)
	2.5	62.3	38.3	
	1.3	146.0	89.7	
	0.9	161.7	99.4	
Standard B^a^	100	0	0	69.7 (67.1–72.4)
	71.4	81.0	49.8	
	51	144.3	88.7	
	36.5	164.7	100	
Negative control material^a^	100	167.0^b^	100	ND^c^
Negative control	0^d^	162.7	ND	ND

^a^Details of Standard A, Standard B, and negative control material are described in the Methods section

^b^There was no significant difference between negative control material and negative control

^c^*ND* not determined

^d^Only medium was added to the plate

### Intradermal administration of the extract in rabbits

Extracts were prepared using non-woven fabric instead of HATS in saline and sesame oil. Each extract was injected at five points in each rabbit. The observation of the skin after 24, 48, and 72 h indicated that the results were all negative. That is, the extract of the non-woven fabric did not induce any irritation.

Three rabbits were used for the intradermal injection of the HATS extracts, and the skin was observed at 24, 48, and 72 h. The extract of HATS, neither in saline nor in sesame oil, showed any irritation. Thus, the extracts from HAT had neither cytotoxic nor inflammation-inducing activity on rabbit skin.

### Skin sensitization test in guinea pigs

The extracts of non-woven fabrics were obtained without HATS, and a skin sensitization test with the extract was conducted using guinea pigs. No positive reactions were observed in any of the conditions except for the positive control, when non-woven fabrics were used.

Then, a skin sensitization test by HATS extract using guinea pigs was conducted. As a result, no skin reaction was observed at any site at 24 and 48 h after patch removal in any animals except for the positive control. Thus, positive reactions were observed only when the positive control (2,4-dinitrochlorobenzene) was used. Regardless of the extraction solvent (saline or sesame oil), the HATS extracts did not induce positive reactions. Indeed, the negative controls (saline control and sesame oil control) did not show positive reactions.

### Administration of HAT to the nasal cavity of rabbits

The HAT suspension was applied once to the nasal cavity of rabbits. The results showed no changes in general condition, body weight, or the conditions of the mucosa at the site of administration throughout the observation period. In the mucosal samples collected after seven or 14 days of administration, no changes were observed by pathological examination of the nasal cavity. In the plasma, no silver or titanium was detected at any of the timepoints of the experiment. These results indicate that under the conditions of this study, HAT powder was not considered to be a primary irritant to the nasal mucosa.

In a separate experiment, HAT was administered daily for 14 or 28 days. Pathological examination of the nasal cavity collected at the end of the 14-day and 28-day treatment periods showed no changes attributable to the administration of the test substances. No silver or titanium was detected in the plasma at any timepoint. Therefore, under the conditions of this study, HAT was not considered to have a cumulative effect as an irritant to the nasal mucosa.

### Intraocular administration study in rabbits

The HAT suspension was administered in the conjunctival sac of the eye of each rabbit. The index of acute ocular irritation was two, and the degree of irritation was classified as non-irritant. No corneal damage was observed in any of the rabbits during the observation period using fluorescein sodium. Therefore, under the conditions of this study, HAT was not considered to be acutely irritating or corrosive to the eyes.

### Oral administration study in rats

In the first experiment, 2000 mg/kg of HAT was administered to five male and five female SD rats, which were observed for 14 days. No abnormalities in general condition or weight were observed. Animal death was not observed, and necropsy after the experiment showed no abnormalities.

In the next experiment, HAT was administered at doses of 0, 30, 100, 300, 1000, and 2000 mg/kg, and no deaths were observed in rats treated with these concentrations. During the study period, no evidence of toxicity related to the test substance was observed in any rat with respect to mean body weight, mean body weight gain, food intake, organ weight, and clinical findings. Gross necropsy revealed no gross lesions associated with the test substance in any of the study groups. Histopathological evaluation indicated no abnormalities associated with the test substance. In conclusion, no toxicity was observed with HAT powder up to a dose of 2000 mg/kg.

### Human patch test using HATS

A total of 25 healthy subjects, 12 males (age 47.2 ± 10.1 years) and 13 females (age 38.4 ± 9.6 years) aged 27–61 years, were subjected to the patch test. Skin lesions were observed and judged at 1 h and 24 h after the removal of the samples. No positive judgment was made for any subject at any timepoint.

## Discussion

Since HATS absorbs and breaks down nasal allergens, masks using HATS are expected to be more effective against nasal allergies than masks using only non-woven fabrics.

In our previous paper, we reported an initial basic study to confirm the safety of HATS by examining the size distribution of HAT particles and their adsorption onto non-woven fabric fibers [1]. However, the present study is the first report of a biological safety study.

There have been reports on the safety of titanium dioxide, silver, and HA as individual substances. For example, many sunscreens use nanoparticles of titanium dioxide, which are considered safe because they are not absorbed [25]. On the other hand, ultrafine titanium dioxide dust has been reported to cause respiratory cancer in rats when exposed to inhalation and intratracheal administration [26]. However, this result can be applied only to subjects exposed to high doses of titanium dioxide, such as workers manufacturing titanium dioxide. To date, human studies have not suggested an association between exposure to titanium dioxide and an increased risk of cancer. The U.S. National Institute for Occupational Safety and Health recommends an exposure limit of 2.4 mg/m^3^ for fine titanium dioxide particles and an exposure limit of 0.3 mg/m^3^ for ultrafine titanium dioxide particles as a time-weighted average concentration for up to 10 h per day in a 40-h work week [27]. We measured the amounts of titanium released from HATS and found that it was considerably lower than the limits (data not shown).

There have also been reports on the safety of silver in humans. Medical applications of silver include wound dressings, creams, and antibiotic coatings for medical devices [28–30]. Wound dressings containing silver sulfadiazine or silver nanomaterials are sometimes used to treat external infections [31–33]. Silver coatings on endotracheal tubes have been used to reduce the incidence of ventilator-associated pneumonia [34]. Silver is generally of low toxicity, and the risk is considered to be negligible when it is used for approved medical applications [35].

On the other hand, HA is a physiological component. Seventy percent of human bone is composed of HA by weight. In addition, it is also a major component of teeth and is sold as an ingredient in toothpaste [36]. Therefore, it is considered safe in many cases, including oral intake. However, there have been no reports on the safety of HAT powder, which is a mixture of titanium dioxide, silver, and HA.

In this study, we conducted mutagenicity tests of HAT extract using bacteria and showed that HAT was not mutagenic. In general, titanium is not water-soluble; therefore, if an effect is observed in the HAT suspension, it is thought to be due to the action of eluted silver or HAT particles. Precipitates were observed in plates containing high concentrations of HAT. Next, we conducted a cytotoxicity test using the extract obtained from HATS using the colony formation method. The results showed moderate cytotoxicity, suggesting that inflammatory reactions may occur in the mucosal tissues.

Based on the results of the above cellular level studies, we conducted in vivo experiments in mammals. Intradermal administration of HATS extract in rabbits showed no irritation. The results of skin sensitization tests on guinea pigs using saline and sesame oil extracts of HATS were negative, indicating that the HATS extracts were not skin sensitizers. Intranasal administration of the HAT powder suspension to rabbits showed no abnormalities. In addition, no silver or titanium was detected in the plasma, and the administration of HAT in the conjunctival sac of rabbits also showed no acute eye irritation/corrosion. Next, we administered HAT powder orally to rats. No toxicity was observed up to a single dose of 2000 mg/kg, and the minimum lethal dose of HAT by oral administration was over 2000 mg/kg.

Finally, the results of a patch test conducted on 25 subjects showed that HAT did not induce irritation reactions.

HATS is used in a mask sandwiched between two sheets of non-woven fabrics that do not contain HAT. Therefore, under normal usage, HATS will not come into contact with human skin. However, if the mask is damaged, it is possible for the HATS to touch the skin. In addition, some HAT particles in the HATS may be exposed through the nose. Therefore, the safety of HAT particles and HATS should be carefully examined.

In this study, HATS was not mutagenic in bacterial tests and was relatively safe when administered to the skin, nasal cavity, and eye mucosa of animals. There were no adverse events, including pathological findings, when administered orally to rats, and neither titanium dioxide nor silver was detected in the plasma after nasal administration. There is a possibility of toxicity if HAT comes into direct contact with cells. However, neither silver nor titanium was detected in the plasma after nasal administration, and no toxicity was observed in dermal, ocular, nasal, or oral administration. Therefore, it is unlikely that HAT comes into direct contact with cells through these routes.

We are currently conducting a clinical trial in Japan under the guidance of the Ministry of Health, Labor, and Welfare to evaluate the safety and efficacy of the drug in patients with allergic rhinitis.

In conclusion, although HAT showed moderate cytotoxicity, in vivo results indicated that HAT is safe because it does not come in direct contact with cells in normal usage, and HATS is safe when used in masks.

## Limitations

This study has the following limitations. First, the experiments were conducted primarily on bacteria, mice, rabbits, guinea pigs, and Chinese hamster cells, and it is not clear whether these results are applicable to humans. Second, although titanium dioxide and silver are insoluble, it is possible that a small amount of dissolution has occurred, and we did not measure the dissolved substances in each experiment. Third, of the hydroxyapatite, silver, and titanium dioxide that make up HAT, hydroxyapatite is a physiological substance and exists in the body in abundance. Therefore, there may be more hydroxyapatite in the body than what was used in the experiment, but its effects have not been studied. Lastly, in our separate study, we showed that HAT reduced Cryj1, a major allergen in cedar pollinosis, and Der f1, a major allergen in dust mite allergy in an in vitro study [1], but whether HATS really reduces nasal allergies such as cedar pollinosis and dust mite allergy needs to be determined by clinical trials.
